# Effectiveness of the Call in Beach Volleyball Attacking Play

**DOI:** 10.2478/hukin-2014-0124

**Published:** 2014-12-30

**Authors:** Stefan Künzell, Florian Schweikart, Daniel Köhn, Olivia Schläppi-Lienhard

**Affiliations:** 1 Institut für Sportwissenschaft, Universität Augsburg, Germany.; 1 Institut für Sportwissenschaft, Universität Bern, Switzerland.

**Keywords:** cooperation, tactics, game observation, communication

## Abstract

In beach volleyball the setter has the opportunity to give her or his hitter a “call”. The call intends that the setter suggests to her or his partner where to place the attack in the opponent’s court. The effectiveness of a call is still unknown. We investigated the women’s and men’s Swiss National Beach Volleyball Championships in 2011 and analyzed 2185 attacks. We found large differences between female and male players. While men called in only 38.4% of attacks, women used calls in 85.5% of attacks. If the male players followed a given call, 63% of the attacks were successful. The success rate of attacks without any call was 55.8% and 47.6% when the call was ignored. These differences were not significant (χ^2^(2) = 4.55, p = 0.103). In women’s beach volleyball, the rate of successful attacks was 61.5% when a call was followed, 35% for attacks without a call, and 42.6% when a call was ignored. The differences were highly significant (χ^2^(2) = 23.42, p < 0.0005). Taking into account the findings of the present study, we suggested that the call was effective in women’s beach volleyball, while its effect in men’s game was unclear. Considering the quality of calls we indicate that there is a significant potential to increase the effectiveness of a call.

## Introduction

In beach volleyball a typical rally consists of a serve by a team A, followed by a serve receive, a set and an attack of a team B. After setting the ball, the setter has the opportunity to observe the opponent’s defense lineup and support her or his hitter by calling a possibly uncovered area in the opponent’s court. If the hitter follows a given call, he or she will hit the ball over the block into the uncovered area. The call is especially helpful in situations where the hitter does not hit a hard driven ball, but tries to win the rally through a well-placed shot. “By definition, the spikes are executed with maximum power and the ball trajectory after hand contact follows a straight line. On the other hand, the shots are relatively softly attacked balls, which are used to place the ball into unprotected areas of the court” (Koch and Tilp, 2009, p. 55).

Beach volleyball is a scientifically well investigated sports discipline. The influence of serve characteristics on performance in men’s and women’s elite beach volleyball has been analyzed (Buscà et al., 2012). Furthermore, psychological factors have been investigated (Kais and Raudsepp, 2004) as well as the influence of expertise in anticipation of different attack shots (Güldenpenning et al., 2013; Cañal-Bruland et al., 2011). Moreover, the effects of the change of the court size have been explored (Palao et al., 2012; Grgantov et al., 2005). However, there is no research to date on the effect of a call in offense.

In the present study, we focused on elite players during matches of the 2011 Swiss Championships and whether the efficiency of the attack was positively influenced by a call.

## Material and Methods

We analyzed 26 matches in the 2011 Swiss Championship in Bern, Switzerland (11 women’s and 15 men’s matches). All matches took place at the Center Court. Participants of the National Championships were the eight best women’s and the twelve best men’s teams of the 2011 Swiss rankings. Four men’s teams and two women’s teams were ranked in the top 50 teams in the world (FIVB rankings). These were classified as elite teams while the other teams were sub-elite teams. All teams played according to a double-out system, which meant that every team was allowed to lose one match, but if they lost a second one they were eliminated of the tournament.

Data was collected by a video camcorder, positioned behind the serving area. A small wireless microphone was interlaced into the net, in order to record all verbal communication between the players on the audio track of the camcorder. Videos were analyzed by two raters to collect the dependent variables.

### Dependent variables

All videos were analyzed with the help of a standardized observation sheet. Two independent observers took note of a multitude of game variables. For the purpose of this paper, variables were confined to “block situation”, “intention of the call”, “quality of the call”, “following the call”, and “success of the attack”. Block situations could be “with block”, “without block”, or “fake block”. A “fake block” is recognized if the blocker stands close to the net, ready for the block and then quickly moves to a defensive position in the court after the opponent’s set. Different teams use different codes for their calls. In “call intention”, individual calls are translated into previously defined values. Possible values for call intentions were “line”, which demands a shot along the sideline next to the hitter, and “dia” that demands a cross-court shot. As well as “cut” demands a shot placed closely behind the net to the opposite sideline and “no-one” which indicates that there is no block at all. An example of the translating process is given here for the case that the setter calls “left”. If the partner hits on the left side of the court, the call “left” has to be translated into “line”. If the hitter is on the right side of the court, the correct decoding of the “left” call is “dia”. Although it is not a usual call, we sometimes observed calls like “with” or “block”. In this case the intention is to inform the partner that there is a block, but without giving a hint about the uncovered spot in the opponent’s court. We denoted these calls as “with block”. Moreover, a lack of a call is denoted. Some calls in the tournament (“line dia dia”) were noted as contradictory. “Following the call” is a dichotome variable. If the hitter plays a shot in the direction of the called target it is denoted as “Yes”, otherwise as “No”. In case of a “no-one” call, a spike is required to follow the call. If a call is contradictory or missing, “following the call” has a missing value as well. There were six possible outcomes which define the “success of the attack” variable. “Perfect” means that the ball hits the sand within the court, “touched” means that the shot is touched by the defending team, but could not be returned, “defended” declares that the rally goes on and “free ball” implies that the ball is easy to pass. “Fault” is denoted if the hitter makes a mistake, and “blocked” if the hitter is blocked. Additionally, the quality of a call was rated. If a call suggests the corner opposite to the defender’s position or her or his actual movement direction, the call was rated as “good”. A call that suggests a spot in the court where the defender is or moves to was rated as “bad”.

## Results

Overall, we analyzed 2185 attacks, 1027 attacks by female athletes (420 elite players, 607 sub-elite players) and 1158 attacks by male athletes (653 elite players, 505 sub-elite players). In men’s competition, 88.2% of attacks forced the opponent to block, in 8.3% no block occurred and in 3.5% a block was faked. In women’s competition, a block was set in 47.7% of attacks, while a fake block was executed in 41.6%, and no block was in 10.6% of the attacks. In 61.6% of the attacks male athletes did not give a call at all, the percentage of attacks without a call in women’s competitions was just 14.5%. 90.5 % of the women’s calls and 87.8% of the men’s calls were rated as good.

Caution is required in analyzing the quantity of the times a call was followed. If a hitter followed her or his partner’s call and placed the shot in the called spot, this could be due to two different reasons. On the one hand, it is possible that he or she understood the given call and followed it. On the other hand, the hitter perhaps intended independently of any call to place a shot in that certain spot. Therefore, the hitter probably had seen the position of the defense player by themselves or it could be for a multitude of other reasons. Video analysis cannot assess this variability and it is not possible to ask the athletes during a competition. Regardless of the reason, we denoted a call as followed whenever the hitter played the ball into the corner that her or his partner had called. This assumption has to be kept in mind during the discussion. The percentage of followed calls was higher for women than for men. While women followed 57.7% of the calls by their partner, male athletes did not even follow half of the calls (46.1 %). Because of the large differences between male and female athletes, more detailed analysis was done separately for each gender.

### Male athletes

The crucial interest of this investigation was the effectiveness of a call. It can be analyzed by comparing the success rate of attacks which followed calls with the success rates of attacks with calls ignored and with no calls. Table 1 shows that when a call was followed, 47.1% of the attacks resulted in kills whereas when a call was ignored (30.1%) or cases without any call (31%) the effectiveness was lower. However, the advantage of following a call diminished when exploring touched balls, which lead to a rally point win for the attacking team. Attacks without a call (24.8%) or an ignored call (17.9%) had higher success rates than attacks with a followed call (15.9%). The reason for this was that a usual attack in men’s beach volleyball, either without a call ora neglected call, was a spike which passed the block. In many cases, it was hit to the defense player, who might touch the ball but was not likely to control it.

**Figure 2:**
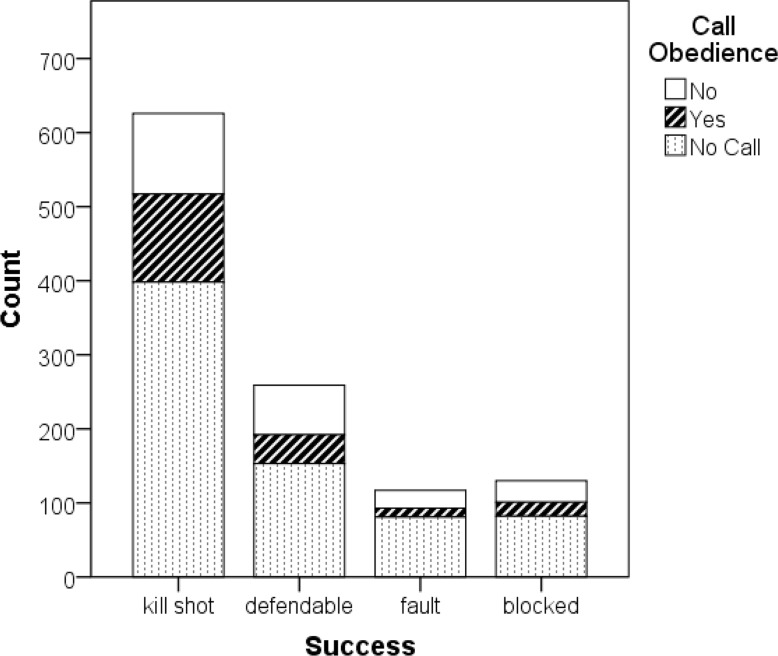
Successful attacks by call obedience in male athletes.

For further analysis we added up the “perfect” and the “touched” category and named them “kill shot”. Moreover, we combined the “defended” and the free ball category. Figure 2 shows that the majority of attacks in the side-out situation, which was the first attack after the serve receive, ended with a kill shot by the receiving team. The percentage of the followed calls within the kill shots (19%) compared to the percentage of calls ignored (17.4%) and the attacks without a call (63.6%) were larger than the percentage within the whole sample (followed call 16.7%; ignored call 20.2%; no call 63.1%). However, these differences were not significant (χ^2^(2) = 4.55, p = .103).

Further analysis compared the elite (top 50 of the FIVB world ranking) with the sub-elite players (Table 2). For both elite (62.1%) and sub-elite (64%) the highest percentage of kill shots was in the situation where they followed a call, followed by situations without any call. The least kills were scored when a call was ignored. The difference between elite and sub-elite player was negligible. Overall, elite players had a slightly better kill shot rate than sub-elite players and less defendable attacks.

### Female athletes

Women gave calls more often than men in beach volleyball (χ^2^(2) = 487.6, p < .0005). When a call was followed, the rate of kills was at 33.7% whereas attacks with ignored calls (22.2%) and without any call (20.7%) had lower rates (Table 3). Contrary to men, this also held for touched balls. So, when we aggregated the categories “perfect” and “touched” to the category “kill shot”, there was a large advantage in favor of attacks where calls were followed. 61.4% of the attacks where a call was followed led directly to a rally win, compared to 42.6% of the attacks where a call was ignored and 35% without a call.

The percentage of the followed calls within the kill shots (59.8%) compared to the percentage of ignored calls (30.8%) and the attacks without a call (9.4%) was larger than the percentage within the whole sample (followed call 49.5%; ignored call 36.8%; no call 13.6%). The differences were highly significant (χ^2^(2) = 23.42, p < .0005).

Women’s call strategy differed with respect to the sports level. Elite female beach volleyball players never called “with block” and omitted a call only in 8.1% of the attacks. Sub-elite female beach volleyball players omitted a call in 18.5% of the attacks and were responsible for all “with block” calls in the survey. Overall, female elite players had a significantly higher rate of kill shots (58.3%) than sub-elite players (45.7%). Further, elite players had a significantly lower rate of defendable attacks (28.8%) than sub-elite players (37.6%) (χ^2^(2) = 12.43, p < 0.0005).

## Discussion

The call strategy of male and female athletes was different. We suppose there are two reasons for this observation. First, the rate of blocked attacks in men’s beach volleyball was far higher than the rate in women’s beach volleyball. Therefore, men very rarely called “no-one” and did not use the “with block” call, because the presence of a block was the usual case. The “line” call intention was the most frequently applied and did not differ in female or male matches. This corresponded to the classical defense formation (Koch and Tilp, 2009) where the blocker tried to cover the line shots, while the defense player tried to cover cross-court attacks. A recent investigation by Seweryniak et al. (2013) of world tournament female beach volleyball competition revealed that this defense formation was chosen in 45% of all defense situations.

The large number of fake blocks corresponded to the high percentage of “no-one” calls in women’s beach volleyball. Since the “with-block” call occurred only in sub-elite female beach volleyball competitions, it seems that this call did not arise from a strategy, but rather an attempt to try to assist the partner.

In women’s beach volleyball, a call seemed to be highly effective. Though we cannot tell if a shot was placed into the uncovered spot because of the call or due to the fact that the hitter perceived the spot herself, the comparison between the no call situation and the call situation gave a clear hint. Women were often more successful with their attacks if their partner gave a call and the hitter placed the ball in the called spot. If the hitter saw the spot by herself and did not need the call, why should the success rate drop when there was no call? A possible explanation is that in some situations the setter might not know what to call and as a consequence called nothing at all. Because of this unclear situation the hitter’s success rate dropped as well. In this case, calls would only be given in clear situations where the hitter saw the opponent’s defensive lineup anyway and a call added nothing to the attacking play. However, in complex situations, where the call would be needed, it would be missing. We cannot rule out this scenario from our data, but we are convinced that world class female beach volleyball players learnt to call particularly in difficult and complex situations. The superiority of elite to sub-elite players in the calling rate supports this notion. Therefore, we concluded that a call was effective in women’s beach volleyball.

In men’s beach volleyball the situation was different. There was a slightly higher success rate if the attacker played the ball to the spot which had been called, but there was no statistical significance. The high rate of attacks without a call reflected the fact that the rate of spikes compared to shots was higher in men’s beach volleyball compared to women’s beach volleyball (Koch and Tilp, 2009).

The question remains why world class athletes sometimes do not follow a call given by their partner. There are four possible reasons which need further investigation. One reason is that hitters may think they know better than their partner. They could pick up information about the location of the opponent’s defense player during the approach. A second reason, presumably prominent in men’s beach volleyball, is that the hitter decides to play a hard driven spike. Thus, the hitter does not care about uncovered spots in the opponent’s court, but rather chooses the direction of the hit according to the opponent’s block. A third reason is that hitters (or their coaches) are afraid that the call reveals the planned attack and the defense player of the opponent team might run soon enough to the called spot. The decision strategies of defense players have been recently investigated. Kredel et al. (2011) were able to show that in a lab situation elite male defense players initiate their movement 250 ms after the ball-hand contact of the opposing hitter. Elite female defense players initiate their movement earlier, about 110 ms after the ball-hand contact. Sub-elite players initiate their movement even earlier. The correctness of the decision was 95% for male elite players and 81% for female elite players. In the lab situation, only visual stimuli could be processed. Kredel et al. (2011) did not consider any auditory stimuli as calls. However, in a qualitative study, Schläppi Lienhard and Hossner (2014) interviewed world class athletes about their decision process in defense situations. Only one out of 19 interviewed athletes mentioned that the opponent’s call influenced his decision. Schläppi-Lienhard and Hossner (2014) concluded that for the defense player the opponent’s call played a minor role at best. Anyway, some internationally successful teams avoid that their call is used by the opponents by calling codes in their (non-English) mother language, which can hardly be decoded by their opponents in time. The fourth reason is that the call perhaps is given too late and therefore could not be followed. The time interval between the call and the ball-hand contact is too short to change the motor plan. The length of this time interval still has to be investigated.

It seems possible that in concern to optimize attacks the quality of calls should be enhanced through practice. Although about 90% of the calls are good calls, there are still about 10% of the calls which suggest a spot in the court where the defense player is or intends to move there. In women’s beach volleyball, 10% wrong calls lead to at least 2 lost points in a set, which can be crucial in a close match.

## Figures and Tables

**Figure 1:**
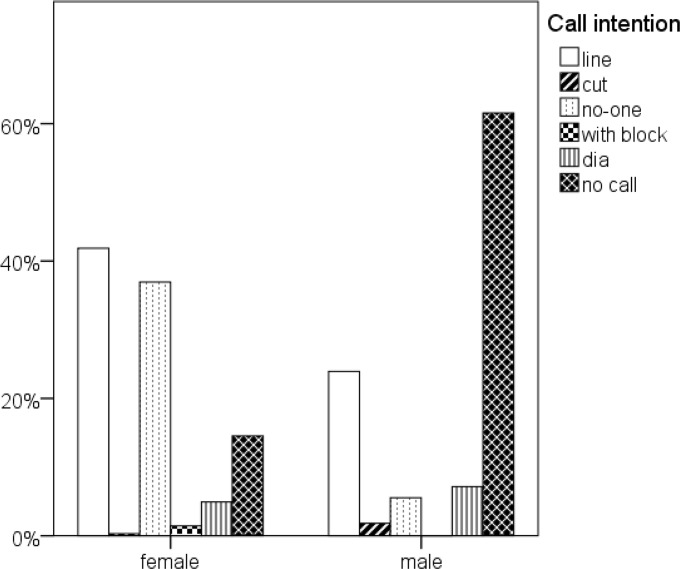
Call intentions by gender. The percentages add up to 100 for each gender

**Figure 3:**
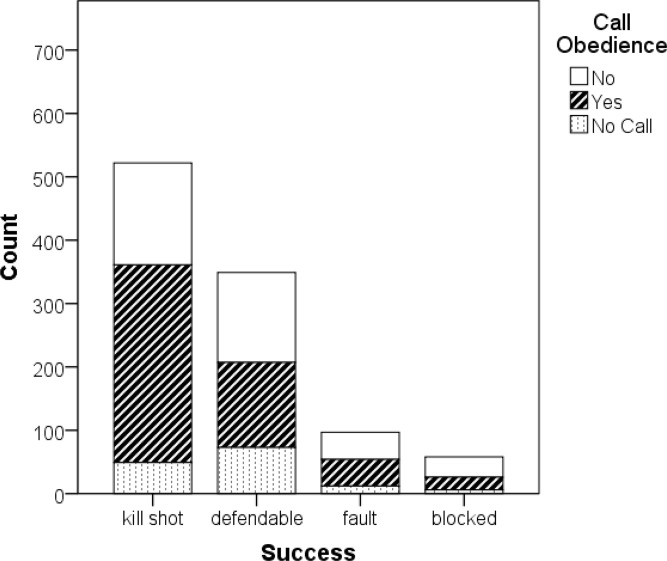
Successful attacks by call obedience in female athletes

**Table 1:** Percentages of successful attacks for male athletes.

		perfect	touched	defended	free ball	fault	blocked
call followed?	No	30.1%	17.5%	27.9%	1.3%	10.5%	12.7%
	Yes	47.1%	15.9%	19.0%	1.6%	6.3%	10.1%

	average with a call	37.8%	16.7%	23.9%	1.4%	8.6%	11.5%
	No call	31.0%	24.8%	19.6%	1.8%	11.3%	11.5%

	overall	33.5%	21.8%	21.2%	1.7%	10.3%	11.5%

Rows add up to 100%. The line “average with a call” includes both the followed and not followed calls.

**Table 2:** The number of attacks broke down by success and following or not following the call for male beach volleyball players

			success			

			kill shot	defendable	fault	blocked
call followed?	No	elite	55	32	16	18
			45.5%	26.4%	13.2%	14.9%
		sub-elite	54	35	8	11
			50.0%	32.4%	7.4%	10.2%
	Yes	elite	64	25	7	7
			62.1%	24.3%	6.8%	6.8%
		sub-elite	55	14	5	12
			64.0%	16.3%	5.8%	14.0%
	No Call	elite	239	83	44	51
			57.3%	19.9%	10.6%	12.2%
		sub-elite	159	70	37	31
			53.5%	23.6%	12.5%	10.4%

	overall	elite	358	140	67	76
			55.9%	21.8%	10.5%	11.9%
		sub-elite	268	119	50	54
			54.6%	24.2%	10.2%	11.0%

Beneath the counts line percentage is denoted, summing up to 100% for every line.

**Table 3:** Percentages of successful attacks for female athletes

		success					

		perfect	touched	defended	free ball	fault	blocked
call followed	No	22.2%	20.4%	34.9%	2.6%	11.4%	8.5%
	Yes	33.7%	27.8%	25.0%	1.4%	8.3%	3.9%

	average with a call	28.8%	24.6%	29.2%	1.9%	9.6%	5.9%
	No call	20.7%	14.3%	30.7%	21.4%	8.6%	4.3%

	overall	27.7%	23.2%	29.4%	4.6%	9.5%	5.7%

Rows add up to 100%. The line “average with a call” includes both the followed and the not followed calls.

**Table 4:** The number of attacks broken down by success and following or not following the call for female beach volleyball players

			success			

			kill shot	defendable	fault	blocked
call followed?	No	elite	82	57	18	12
			48.5%	33.7%	10.7%	7.1%
		sub-elite	79	85	25	20
			37.8%	40.7%	12.0%	9.6%
	Yes	elite	150	47	15	6
			68.8%	21.6%	6.9%	2.8%
		sub-elite	162	87	27	14
			55.9%	30.0%	9.3%	4.8%
	No call	elite	13	17	3	0
			39.4%	51.5%	9.1%	0.0%
		sub-elite	36	56	9	6
			33.6%	52.3%	8.4%	5.6%

	overall	elite	245	121	36	18
			58.3%	28.8%	8.6%	4.3%
		sub-elite	277	228	61	40
			45.7%	37.6%	10.1%	6.6%

Beneath the counts line percentage is denoted, summing up to 100% for every line.
